# Alterations of Resting-State Static and Dynamic Functional Connectivity of the Dorsolateral Prefrontal Cortex in Subjects with Internet Gaming Disorder

**DOI:** 10.3389/fnhum.2018.00041

**Published:** 2018-02-06

**Authors:** Xu Han, Xiaowei Wu, Yao Wang, Yawen Sun, Weina Ding, Mengqiu Cao, Yasong Du, Fuchun Lin, Yan Zhou

**Affiliations:** ^1^Department of Radiology, Ren Ji Hospital, School of Medicine, Shanghai Jiao Tong University, Shanghai, China; ^2^Department of Child and Adolescent Psychiatry, Shanghai Mental Health Center, Shanghai Jiao Tong University, Shanghai, China; ^3^National Center for Magnetic Resonance in Wuhan, State Key Laboratory of Magnetic Resonance and Atomic and Molecular Physics, Wuhan Institute of Physics and Mathematics, Chinese Academy of Sciences, Wuhan, China

**Keywords:** functional magnetic resonance imaging, internet gaming disorder, resting-state, functional connectivity, dorsolateral prefrontal cortex

## Abstract

Internet gaming disorder (IGD), a major behavior disorder, has gained increasing attention. Recent studies indicate altered resting-state static functional connectivity (FC) of the dorsolateral prefrontal cortex (DLPFC) in subjects with IGD. Whereas static FC often provides information on functional changes in subjects with IGD, investigations of temporal changes in FC between the DLPFC and the other brain regions may shed light on the dynamic characteristics of brain function associated with IGD. Thirty subjects with IGD and 30 healthy controls (HCs) matched for age, gender and education status were recruited. Using the bilateral DLPFC as seeds, static FC and dynamic FC maps were calculated and compared between groups. Correlations between alterations in static FC and dynamic FC and clinical variables were also investigated within the IGD group. The IGD group showed significantly lower static FC between the right DLPFC and the left rolandic operculum while higher static FC between the right DLPFC and the left pars triangularis when compared to HCs. The IGD group also had significantly decreased dynamic FC between the right DLPFC and the left insula, right putamen and left precentral gyrus, and increased dynamic FC in the left precuneus. Moreover, the dynamic FC between the right DLPFC and the left insula was negatively correlated with the severity of IGD. Dynamic FC can be used as a powerful supplement to static FC, helping us obtain a more comprehensive understanding of large-scale brain network activity in IGD and put forward new ideas for behavioral intervention therapy for it.

## Introduction

Internet addiction disorder (IAD) is defined as a failure to control one’s impulses to excessively use the Internet, which may eventually lead to serious negative consequences, such as sleep insufficiency, poor work performance, and loss of interest in other activities (Young and Rogers, 1998). Internet gaming disorder (IGD) is the most common subtype (57.5%) of IAD (Kishi et al., 2009) and has become a serious mental health issue around the world, thereby requiring additional investigation, as exemplified by its inclusion in Section III of the Diagnostic and Statistical Manual of Mental Disorders, 5th Edition (DSM-V; American Psychiatric Association, 2013). Adolescence, as a developmental stage, seems more vulnerable to IGD because of the immature cognitive control during this period (Pine et al., 2001; Silveri et al., 2004) and IGD among adolescents has become an important public concern around the world, e.g., in Asia, Europe and the USA (Cao et al., 2011; Christakis et al., 2011; Durkee et al., 2012). However, due to a lack of adequate comprehensive findings, the precise pathogenesis of IGD remains unclear.

A growing body of evidence shows that distributed neural circuits exhibit spontaneous activity at rest (Ge et al., 2017; Yuan et al., 2017). These slow-frequency fluctuations are temporally correlated within spatially distinct but functionally related networks. Resting-state functional connectivity (FC) has revealed several networks that are consistently found in healthy subjects and patients with neuropsychiatric diseases (Damoiseaux et al., 2006; Liu et al., 2015a; Chen et al., 2017). Evaluation of the resting-state FC provides an opportunity to explore functional abnormalities in IGD. For example, Lin et al. (2015) found reduced connectivity between the nucleus accumbens and the caudate head, which implied alterations in reward-related functions in IGD, indicating people addicted to the Internet may prefer to select smaller immediate rewards rather than larger rewards that occur in the future, such as good health, good relationships or occupational success. A significantly higher amygdala-insula FC, which contributes to emotional regulation, was detected in a previous study, suggesting that subjects with IGD might continue to participate in online gaming to escape from negative emotional experiences (Ko et al., 2015). Furthermore, Zhang J. T. et al. (2016) reported diminished connectivity between the insula and the dorsolateral prefrontal cortex (DLPFC), which would therefore weaken the capacity to integrate information and may be related to the diminished cognitive control that leads to persistent engagement in IGD. Specific neurobiological networks have been shown by resting-state FC to reveal the possible mechanisms of IGD.

The clinical characteristics of IGD include craving and over-engagement despite the negative consequences of participation in internet game playing. Therefore, the key feature of IGD is lost or diminished self-control, which is a cognitive process that is necessary for regulating one’s behavior (Dong et al., 2015). Executive functions including basic cognitive processes such as attentional control, inhibitory control, working memory, and cognitive flexibility, enable individuals to inhibit their desires and limit participation in hedonic behaviors under unfavorable circumstances (Dong et al., 2013). The core area governing executive function is the DLPFC (Kaplan et al., 2016). Structural abnormalities of the DLPFC have been commonly observed in IGD (Lin et al., 2012), and abnormal brain FC of the DLPFC has also been reported (Ko et al., 2013). Han et al. (2015) demonstrated that both IGD and alcohol addiction (AD) participants had positive FC between the DLPFC, cingulate, and cerebellum, indicating that IGD and AD participants may share deficits in executive function. Furthermore, the brain activity of the DLPFC was shown to be positively correlated with cravings in response to online game cues in IGD subjects (Ko et al., 2009).

However, the aforementioned resting-state FC studies used the blood-oxygen-level dependent (BOLD) signal over the entire scan (with a typical duration of 5–10 min), assuming that the resting-state FC is stationary. Recently increasing evidence has suggested that resting-state FC is dynamic and exhibits significant spontaneous fluctuations on a smaller time scale (Liu and Duyn, 2013; Calhoun et al., 2014; Di and Biswal, 2015; Preti et al., 2017). Dynamic FC has been widely used in a lot of fields, such as idiopathic generalized epilepsy, schizophrenia, major depressive disorder, chronic fatigue syndrome, long-term sleep deprivation and chronic headache patients (Glerean et al., 2012; Demirtaş et al., 2016; Wang et al., 2016, 2017; Liu F. et al., 2017; Xu et al., 2017; Boissoneault et al., 2018), as well as being used to examine individual time-varying properties of the chronnectome (Liu et al., 2018). The dynamic FC derived from resting-state FC functional magnetic resonance imaging (fMRI) data is expected to reveal meaningful information about IGD related brain functional impairments that may be lost when longer time scales or entire scans are used, which would extend the understanding of the effects of IGD on the brain’s functional organization. Since sliding-window analysis as the traditional method has received critical controversy (Wang et al., 2016), and the flexible least square (FLS) method is a distribution-free approach to estimate the continuous changes in model parameters at each observation which could avoid the trouble of choosing parameters (Hutchison et al., 2013), we applied the FLS method in the present study. Investigating time-varying FC will be helpful to deepen our understanding of the neural mechanisms subserving mental disorders, especially from the aspect of temporal stability.

Therefore, the present study assessed both static and dynamic FC with the use of resting-state fMRI data. The bilateral DLPFC were chosen as seeds based on previous research (Zhou et al., 2007). We hypothesized that FC based on the bilateral DLPFC would show impaired engagement in subjects with IGD, reflecting aberrant static and dynamic FC patterns relative to the healthy controls (HCs).

## Materials and Methods

### Participants

The study was approved by the Research Ethics Committee of Ren Ji Hospital, School of Medicine, Shanghai Jiao Tong University, China. All participants were informed of the aims of our study before the MRI examination. Full and written informed consent was obtained from each subject.

Thirty right-handed youths with IGD and 30 right-handed, age-, gender- and education-matched HCs participated in the present study. The diagnostic questionnaire for IGD subjects was adapted from the modified Diagnostic Questionnaire for Internet Addiction (i.e., the YDQ) criteria by Beard and Wolf (2001). All participants underwent a simple physical examination including blood pressure and heart rate measurements and were interviewed by a psychiatrist regarding their medical history of nervous, motor, digestive, respiratory, circulatory, endocrine, urinary and reproductive problems. The exclusion criteria included the following: (1) a history of substance abuse and major psychiatric disorders, such as schizophrenia, depression, anxiety disorder, or psychotic episodes; (2) hospitalization for psychiatric disorders; and (3) MRI contraindications.

Four questionnaires were used to assess patients’ clinical features, namely, the Chen Internet Addiction Scale (CIAS), Self-Rating Anxiety Scale (SAS), Self-Rating Depression Scale (SDS), and Barratt Impulsiveness Scale-11 (BIS-11). The CIAS contains 26 items on a four-point Likert scale, and represents the severity of internet addiction. All questionnaires were initially written in English then translated into Chinese.

### MRI Acquisition

MRI examinations were conducted by using a 3.0T MRI scanner (Signa HDxt, GE Medical System, Milwaukee, WI, USA) as described in our previous publication (Wang et al., 2015). A standard head coil was used, and the head was fixed with a foam pad. The participants were instructed to keep their eyes closed, remain motionless, stay awake, and not to think of anything during resting-state fMRI. Each fMRI scan lasted 440 s. A gradient-echo echo-planar sequence was used in functional imaging. Thirty-four transverse slices (repetition time [TR] = 2000 ms, echo time [TE] = 30 ms, field of view [FOV] = 230 × 230 mm^2^, 3.6 × 3.6 × 4 mm^3^ voxel size) aligned along the anterior commissure-posterior commissure line were acquired. Several other sequences were acquired including the following: (1) 3D Fast Spoiled Gradient Recalled sequence (3D-FSPGR) images (TR = 6.1 ms, TE = 2.8 ms, TI = 450 ms, slice thickness = 1 mm, gap = 0, flip angle = 15°, FOV = 256 × 256 mm^2^, number of slices = 166, 1 × 1 × 1 mm^3^ voxel size); (2) axial T1-weighted fast spin-echo sequence (TR = 1725 ms, TE = 24 ms, FOV = 256 × 256 mm^2^, 34 slices, 0.5 × 0.5 × 4 mm^3^ voxel size); and (3) axial T2-weighted fast spin-echo sequence (TR = 9000 ms, TE = 120 ms, FOV = 256 × 256 mm^2^, 34 slices, 0.5 × 0.5 × 4 mm^3^ voxel size).

### Data Processing

Both axial T1- and T2-weighted images were inspected by two experienced neuroradiologists and no gross abnormalities were observed in any participant. Data were analyzed using DPABI version 2.0^1^ and SPM8^2^ software packages. The first 10 images were excluded to ensure steady state, and the remaining 210 images were processed. The images were then corrected for slice timing and realigned to the first image for head movement correction. The data with movement greater than 1 mm maximum translation in x, y, or z or 1° of maximum rotation about the three axes were discarded. No participant was excluded due to excessive head motion. Afterward, the functional images were normalized to the standard Montreal Neurological Institute (MNI) space. The normalized volumes were resampled to a voxel size of 3 mm × 3 mm × 3 mm and spatially smoothed using an isotropic Gaussian filter of 6 mm full width at half maximum. Further preprocessing included the removal of linear trends and temporal bandpass filtering (0.01–0.08 Hz) to reduce low-frequency drifts as well as high-frequency respiratory and cardiac noise. Possible contamination from several nuisance signals, including the signals of white matter, cerebral spinal fluid, global signal, and six motion vectors, were regressed out before FC analysis. We computed the mean framewise displacement (FD) by averaging the FD of each subject from each time point and found no group difference between IGD and HC group (*p* = 0.24). Then each participant’s mean FD was included in group-level analyses as a covariate.

#### Definition of Seed Regions

The two seed regions (right DLPFC and left DLPFC) were generated using the free software WFU_PickAtlas^3^ (Maldjian et al., 2003) which has been used in previous studies (Schon et al., 2005). In the present study, the bilateral DLPFC refers to the bilateral Brodmann’s area 46 (BA46) in the middle frontal gyrus.

#### Functional Connectivity Analysis

For the static FC analysis, a reference time course for each seed (right DLPFC and left DLPFC) was obtained by averaging the time series of all voxels in the ROI. Then, a correlation map was obtained by computing the correlation coefficients between the reference time series and the time series of the whole-brain voxels. Correlation coefficients were then converted to Z-values using the Fisher Z-transformation to improve the normality of the distribution (Wang et al., 2006; Liu et al., 2015b). The individual Z-scores were entered into SPM8 for a random-effects one-sample *t*-test to determine brain regions with significant connectivity to the left DLPFC and right DLPFC within each group (*p* < 0.001, FDR-corrected). Then, these individual Z-scores were entered into SPM8 for a two-sample* t*-test to identify regions showing significant differences with each seed region between the IGD group and HC group. The results of static FC were identified for thresholds at *p* < 0.05, a minimum cluster size of 37 voxels (AlphaSim-corrected, with parameters including the following: single voxel *p* = 0.001; 5000 simulations; a mean estimated spatial correlation of 9.06 mm × 8.29 mm × 7.72 mm FWHM; and the mask of global gray matter). Regions with statistical significance were masked on MNI brain templates.

The dynamic FC analysis was performed with the dynamic brain connectivity (Dynamic BC) toolbox (Liao et al., 2014). A time-varying parameter regression equation was employed to describe the dynamic interactions between brain regions: y(t) = x(t)β(t) + u(t), where x(t) and y(t) represent the variables of each seed and target region respectively, u(t) represents the approximation error, and β(t) represents the coefficient reflecting the dynamic connectivity between the variables *x* and *y* at time *t*. We employed the FLS method in this study to explore the dynamic FC between the bilateral DLPFC and all other regions of the brain. For the FC coefficients of all the time points, we estimated the dynamic FC-variance to represent the time-varying dynamic FC term. The results of dynamic FC were identified for thresholds at *p* < 0.05, a minimum cluster size of 20 voxels (AlphaSim-corrected with parameters including the following: single voxel *p* = 0.001; 5000 simulations; a mean estimated spatial correlation of 7.05 mm × 6.83 mm × 6.12 mm FWHM; and the mask of global gray matter). Regions with statistical significance were masked on MNI brain templates.

For group comparisons of demographic and clinical measures, two-sample *t*-tests were performed using SPSS version 19.0 (IBM Corporation, Armonk, NY, USA), and a chi-square test was used for gender comparisons. SPSS was used to analyze the relationship between the severity of IGD and the strength of FC (static FC and dynamic FC). Linear correlational analyses were performed between the clinical measurements and the mean Z-score and dynamic FC-variance of each cluster showing significant group differences. A two-tailed* p*-value of 0.05 was considered statistically significant for the analyses conducted in SPSS.

We further examined whether the relationship between static FC and dynamic FC was altered in IGD subjects. To this end, we calculated Pearson’s correlation between static FC and dynamic FC maps for each subject. Correlation coefficients were then converted to Z-values using the Fisher Z-transformation and two-sample *t*-test was performed to compare the relationship between GD group and HC group.

## Results

### Demographic and Clinical Results

There were no significant differences in the distributions of age, gender and years of education between the IGD group and the HC group. The IGD subjects showed significantly longer duration of internet use per week and higher CIAS, BIS-II, and SDS scores than HCs (all *p*s <0.001). No differences in SAS scores were found between the two groups (Table 1).

**Table 1:** Demographic and behavioral characteristics of the included participants.

	HC group (*n* = 30) (Mean ± SD)	IGD group (*n* = 30) (Mean ± SD)	*p* value
Age (years)	20.83 ± 2.90	21.20 ± 2.66	0.61
Gender (M/F)	13/17	12/18	0.793
Education (years)	11.80 ± 2.17	10.80 ± 1.62	0.48
Time for internet use per week (hours)	9.80 ± 8.80	36.87 ± 18.53	<0.001
Chen internet addiction scale (CIAS)	41.60 ± 9.39	74.70 ± 9.03	<0.001
Self-rating anxiety scale (SAS)	46.37 ± 10.41	49.03 ± 8.36	0.27
Self-rating depression scale (SDS)	41.33 ± 8.64	52.87 ± 9.39	<0.001
Barratt impulsiveness scale-11 (BIS-11)	56.25 ± 7.07	62.53 ± 7.12	<0.001

*Abbreviation: SD, standard deviation; IGD, internet gaming disorder; HC, healthy control. Two-sample t test was used for group comparisons and chi-square was used for gender comparison*.

### Differences in Static and Dynamic Functional Connectivity

**Figure 1:**
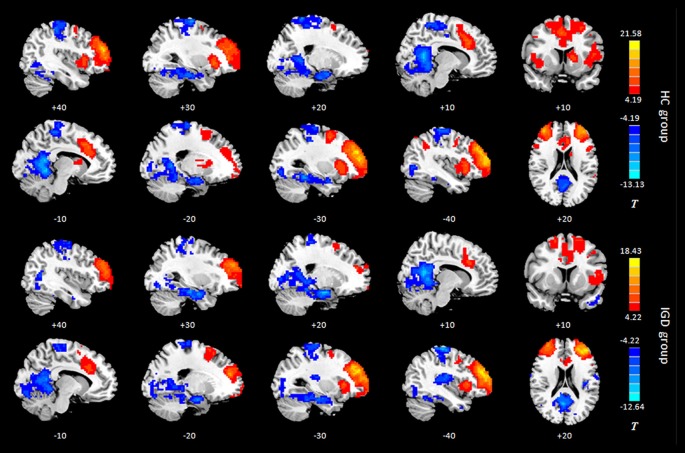
Intragroup maps of connectivity to the left DLPFC of the resting-state networks. Significant connectivity regions are overlaid on the MNI template (*p* < 0.001, FDR-corrected). Color bar, yellow (orange): positive functional connectivity (FC), blue: negative FC. HC, healthy control; IGD, internet gaming disorder; DLPFC, dorsolateral prefrontal cortex; MNI, Montreal Neurological Institute.

**Figure 2:**
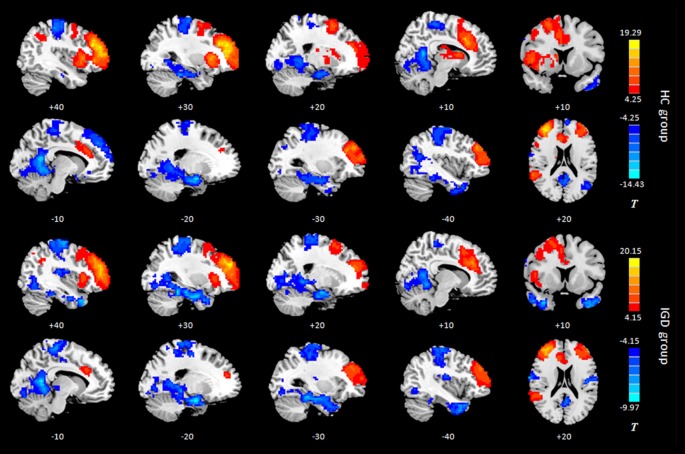
Intragroup maps of connectivity to the right DLPFC of the resting-state networks. Significant connectivity regions are overlaid on the MNI template (*p* < 0.001, FDR-corrected). Color bar, yellow (orange): regions showing positive FC, blue: regions showing negative FC. HC, healthy control; IGD, internet gaming disorder. DLPFC, dorsolateral prefrontal cortex; MNI, Montreal Neurological Institute.

**Figure 3:**
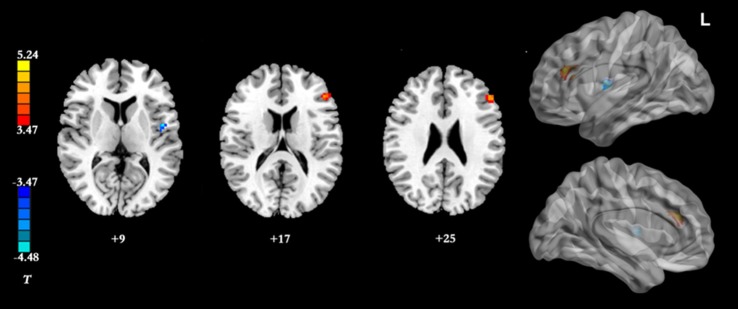
Brain regions showing group differences between the IGD group and HC group in resting-state static FC with the right DLPFC as seed ROI (*p* < 0.05, AlphaSim-corrected). Color bar, yellow (orange): regions showing significantly higher static FC, blue: regions showing significantly lower static FC. IGD, internet gaming disorder. HC, healthy control; DLPFC, dorsolateral prefrontal cortex; FC, functional connectivity.

**Figure 4:**
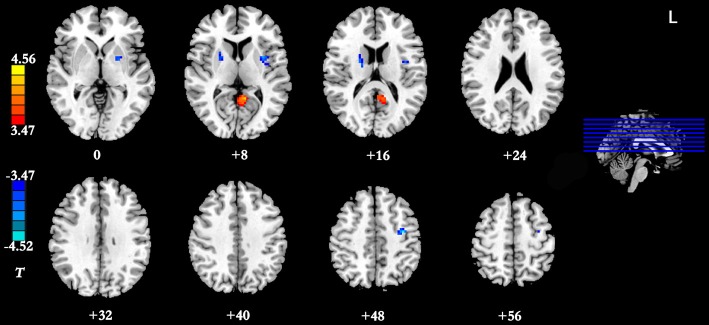
Brain regions showing group differences between the IGD group and the HC group in dynamic FC with the right DLPFC as seed ROI (*p* < 0.05, AlphaSim-corrected). Color bar, yellow (orange): regions showing significantly increased dynamic FC-variance, blue: regions showing significantly decreased dynamic FC-variance. IGD, internet gaming disorder; HC, healthy control; DLPFC, dorsolateral prefrontal cortex; FC, functional connectivity.

Results of the within-group analysis are shown in Table 2, Figures 1, 2. In the between-group analysis, the IGD group showed significantly lower static FC between the right DLPFC and the left rolandic operculum and significantly higher static FC between the right DLPFC and the left pars triangularis compared with the HC group. Meanwhile, the IGD group showed significantly decreased dynamic FC between the right DLPFC and the left insula, the right putamen and the left precentral gyrus and significantly increased dynamic FC-variance with the left precuneus compared with the HC group. No significant differences were seen between the left DLPFC and other brain regions, either in static or dynamic FC (Table 3, Figures 3, 4).

**Table 2:** Brain connectivity in internet gaming disorder (IGD) group and healthy control (HC) group.

Peak MNI coordinate region	Peak MNI coordinates			Number of cluster voxels	Peak *t* value
	*x*	*y*	*z*
**The left DLPFC_HC group**					
Left Middle frontal gyrus (BA46)	−30	48	27	1987	21.57
Left Superior frontal gyrus, medial (BA32)	0	18	42	1141	15.24
Left SupraMarginal (BA48)	−54	−45	33	426	10.24
Right Lingual (BA30)	6	−54	9	2487	−13.13
Right Precentral (BA6)	27	−15	72	1930	−9.31
Right Middle frontal gyrus (BA46)	39	51	18	1453	18.8
**The left DLPFC_IGD group**					
Left Middle frontal gyrus (BA46)	−42	48	15	1758	18.43
Left Superior frontal gyrus, medial (BA32)	−3	24	42	864	10.2
Left Postcentral/Precentral (BA4)	−48	−15	60	731	−12.65
Left Insula (BA48)	−36	−18	15	577	−8.18
Right Lingual (BA17)	6	−60	9	3169	−11.43
Right Middle frontal gyrus (BA46)	33	54	24	887	13.59
Right Precentral (BA4)	57	−9	48	594	−7.83
**The right DLPFC_ HC group**					
Left Middle frontal gyrus (BA46)	−30	48	30	707	13.62
Left Superior frontal gyrus, medial (BA9)	−9	45	39	394	−9.52
Left Middle temporal gyrus (BA21)	−57	−9	−15	427	−8.41
Left Hippocampus (BA28)	−21	−6	−21	2784	−14.43
Right Middle frontal gyrus (BA46)	33	36	30	2363	19.29
Right Postcentral (BA3/4)	54	−12	54	2070	−11.01
Right Median cingulate and paracingulate gyri (BA24)	6	18	36	1100	16.44
Right SupraMarginal (BA40)	57	−42	36	701	13.1
**The right DLPFC_ IGD group**					
Left Middle frontal gyrus (BA46)	−45	51	12	835	13.94
Left Hippocampus (BA36)	−24	−9	−24	2231	−9.97
Right Postcentral (BA43)	69	−6	24	2655	−9.14
Right Middle frontal gyrus (BA46)	30	51	27	1902	20.15
Right Anterior cingulate and paracingulate gyri (BA32)	9	29	28	869	13.14
Right SupraMarginal (BA48)	54	−42	30	711	14.26
Right Inferior frontal gyrus, opercular part (BA48)	51	15	9	406	10.62

*Abbreviation: MNI, Montreal Neurological Institute; IGD, internet gaming disorder; BA, Brodmann area; DLPFC, dorsolateral prefrontal cortex; HC, healthy control; FC, functional connectivity. Note: t < 0 indicates negative connectivity (p < 0.001, FDR-corrected)*.

**Table 3:** Regions showing group differences in static functional connectivity (FC) and dynamic FC with the right dorsolateral prefrontal cortex (DLPFC).

Peak MNI coordinate region	Peak MNI coordinates			Number of cluster voxels	Peak * t* value
	*x*	*y*	*z*
Static FC					
Left rolandic operculum (BA48)	−48	−3	9	40	−4.36
Left pars triangularis (BA45)	−51	33	21	67	5.24
Dynamic FC-variance					
Left insula (BA48)	−33	3	9	49	−3.86
Left precuneus (BA30)	−9	−51	12	59	4.13
Right putamen	21	0	12	33	−3.58
Left precentral gyrus (BA6)	−36	−9	48	33	−4.45

*Abbreviation: MNI, Montreal Neurological Institute; IGD, internet gaming disorder; BA, Brodmann area; DLPFC, dorsolateral prefrontal cortex; HC, healthy control; FC, functional connectivity. Note: t < 0 indicates IGD group < HC group (p < 0.05, AlphaSim-corrected)*.

### Pearson’s Correlational Analysis

**Figure 5:**
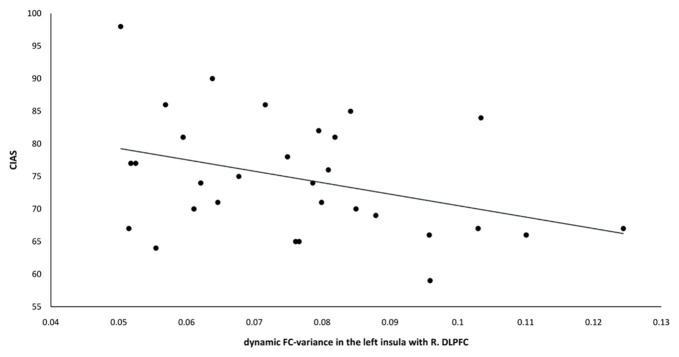
The dynamic FC-variance between the left insula and right DLPFC was negatively correlated with the CIAS scores of the IGD group (*r* = −0.369, *p* = 0.045). FC, functional connectivity; DLPFC, dorsolateral prefrontal cortex; CIAS, Chen Internet Addiction Scale; IGD, internet gaming disorder.

A significant negative correlation was observed between the dynamic FC-variance of right DLPFC and left insula and the severity of IGD (*r* = −0.369, *p* = 0.045; Figure 5). However, no significant difference (*p* = 0.67) in the relationship between static FC and dynamic FC was observed between the IGD group (*r* = −0.05 ± 0.06) and the HC group (*r* = −0.06 ± 0.08).

## Discussion

The present study presents a preliminary analysis of bilateral DLPFC FC by combining static FC and dynamic FC analyses in IGD subjects. This study first found that subjects with IGD had significantly lower static FC between the right DLPFC and the left rolandic operculum but higher between the right DLPFC and the left pars triangularis compared with the HCs. Our results also demonstrated that subjects with IGD showed significantly decreased dynamic FC-variance between the right DLPFC and the left insula, the right putamen and the left precentral gyrus but increased with the left precuneus compared with the HC group. In addition, the dynamic FC-variance of the right DLPFC and left insula was negatively correlated with the severity of IGD.

Based on previous studies, both functional and structural abnormalities of the DLPFC have been commonly observed in IGD (Yuan et al., 2011, 2017; Du et al., 2017). Complex cognitive functions, such as conflict-induced behavioral adjustment, attention, working memory, and inhibitory control, have usually been associated with activations in DLPFC. In addition, functional neuroimaging studies have revealed that the DLPFC is abnormally activated in response to internet gaming-related stimuli in the IGD group and have concluded that IGD subjects must employ more cognitive resources to check their erroneous behaviors during tasks (Zhang et al., 2016a). Han et al. (2015) demonstrated that the IGD group showed similar DLPFC FC as the alcohol-dependent group, which indicates that IGD subjects and alcohol-dependent subjects may share similar deficits in executive function. IGD is different from alcohol-dependency in that no chemical or substance intake is involved; however, excessive internet use may lead to physical dependence similar to that observed in other addictions. Our results showed that the subjects with IGD had significantly lower static FC between the right DLPFC and the left rolandic operculum, and significantly higher static FC between the right DLPFC and the left pars triangularis compared with the HCs. The pars triangularis and rolandic operculum are both implicated in the semantic processing of language (Demb et al., 1995; Behroozmand et al., 2015). The abnormal resting-state FC of the right DLPFC with these two regions in the IGD group may represent an impairment of the speech system, and more research is needed to explain this in the future.

The current study also demonstrated that the IGD group showed significantly decreased dynamic FC-variance between the right DLPFC and the left insula, the right putamen and the left precentral gyrus, and significantly increased dynamic FC-variance with the left precuneus. The greater the variance, the more frequent the switching of the FC strength between the two regions. Zhang et al. (2016b) found that the IGD is associated with an altered prefrontal-insular network. The insula has been implicated in playing a role in addiction, as a neural substrate for addictive urges (Naqvi et al., 2007). Whole-brain analyses have indicated that individuals with IGD showed decreased gaming cue-induced brain activation within regions that included the insula (Liu L. et al., 2017). Therefore, the decreased dynamic FC-variance between the right DLPFC and the left insula (reduced switching frequency) may relate to the neural substrate for addictive urges and behaviors of the IGD.

FitzGerald et al. (2012) reported that action-specific valuation processes are encoded in brain regions including the putamen and insula, consistent with the key role suggested for these regions in linking rewards to goal-directed or habitual actions. The putamen, a part of the dorsal striatum, is a brain region associated with cognitive processing, more specifically, the putamen has been associated with the control of habitual behaviors (Hong et al., 2015). The significantly decreased dynamic FC-variance between the right DLPFC and the right putamen may relate to the habitual goal-directed behaviors involved in the process of game upgrading. A large body of previous research on substance addiction highlights the dorsal striatum as a core subcortical region involved in craving, compulsive drug seeking and drug taking (Koob and Volkow, 2010). In a comparison study between participants with pediatric bipolar disorder and ADHD (Passarotti et al., 2010), increased DLPFC activity and decreased activation of the striatal areas were observed, and this mismatch is thought to be caused by the immaturity of the striatal circuitry, including that of the DLPFC. Our finding of a significantly decreased dynamic FC-variance between the right DLPFC and the right putamen is similar to previous finding. Meanwhile, a significant negative correlation was observed between the dynamic FC-variance of the right DLPFC and the left insula and the severity of IGD. Taken together, these findings suggest that a large-scale brain network including the right DLPFC, insula and putamen is important for the development of IGD.

In the present study, the IGD group showed a significantly decreased dynamic FC-variance between the right DLPFC and left precentral gyrus compared with the HC group. The postcentral gyrus belongs to the somatosensory and sensorimotor cortices, and the maladaptive interactions between the postcentral gyrus and the posterior insula among IGD subjects may reflect abnormalities in receiving, processing, and integrating body-relevant signals to guide ongoing behavior (Paulus and Stewart, 2014). While playing internet games, the IGD subjects need to attend to sounds and control their avatars skillfully to dodge enemies or select weapons (Bavelier et al., 2011). In a previous study, Zhang et al. (2011) found activation of the precuneus in the IGD group. Coupled with this result, the significantly increased dynamic FC-variance between the right DLPFC and the left precuneus observed in the present study suggests that additional studies are needed to investigate the abnormal FC between the right DLPFC and the left precuneus of IGD subjects. We found no significant between-group difference in the relationship between static FC and dynamic FC, further research should be performed to help understand the consistent and inconsistent results observed between static FC and dynamic FC analysis.

Taken together, these findings demonstrate that the IGD group exhibits abnormal dynamic FC; these dynamics provide additional information about the state of functional circuits and are sensitive measures in detecting changes in the brain circuits of IGD subjects. Moreover, an improved understanding of the link between IGD and dynamics can further enhance our understanding of how dynamic network properties support normal brain function and contribute to the development of IGD.

Several limitations of the current study should be considered. First, in the dynamic FC analysis of the present study, the only parameter discussed is the dynamic FC-variance, and we expect further studies to focus on combining more parameters and clarifying the physiological mechanisms of dynamic FC in subjects with IGD. Second, the current study only analyses the changes in FC and ignores the structural alterations of brain regions as well as the causal relationship between structural alterations and FC changes. Another limitation is that no significant correlation remained after Bonferroni correlation (*p* = 0.002 (0.05/24)), which may be related to the small sample size. In addition, we found abnormalities in the FC of some brain regions and linked these to relevant function impairments; however, this needs to be further confirmed in the future. Finally, these findings are cross-sectional and longitudinal studies are needed to determine whether altered right DLPFC connectivity precedes the development of IGD or is the consequence of excessive gaming (Zhang J. T. et al., 2016).

In conclusion, the present study is the first to assess altered FC of the bilateral DLPFC in IGD subjects using both static FC and dynamic FC. Dynamic FC can be used as a powerful supplement to static FC, helping us to obtain a more comprehensive understanding of large-scale brain network activity in IGD and reveal new ideas for behavioral intervention therapy for IGD.

## Author Contributions

YD and YZ were responsible for the study concept and design. XH, XW, YW, YS, WD and MC contributed to the acquisition of MRI data. XH, XW, YW and YS performed the data analysis and assisted with interpretation of findings. XH and XW drafted the manuscript. FL and YZ provided critical revision of the manuscript for important intellectual content. All authors critically reviewed the content and approved the final version for publication.

## Conflict of Interest Statement

The authors declare that the research was conducted in the absence of any commercial or financial relationships that could be construed as a potential conflict of interest.

## Footnotes

^1^http://rfmri.org/dpabi

^2^http://www.fil.ion.ucl.ac.uk/spm

^3^http://www.ansir.wfubmc.edu
